# Supplementary material for: Fabrication of sharp silicon arrays to wound Caenorhabditis elegans
Source: Sci Rep. 2020 Feb 27;10:3581. doi: 10.1038/s41598-020-60333-7 (PMC7046703; doi:10.1038/s41598-020-60333-7)
Supplement: Supplementary file 1 — Supplementary Information. [file 41598_2020_60333_MOESM1_ESM.pdf]

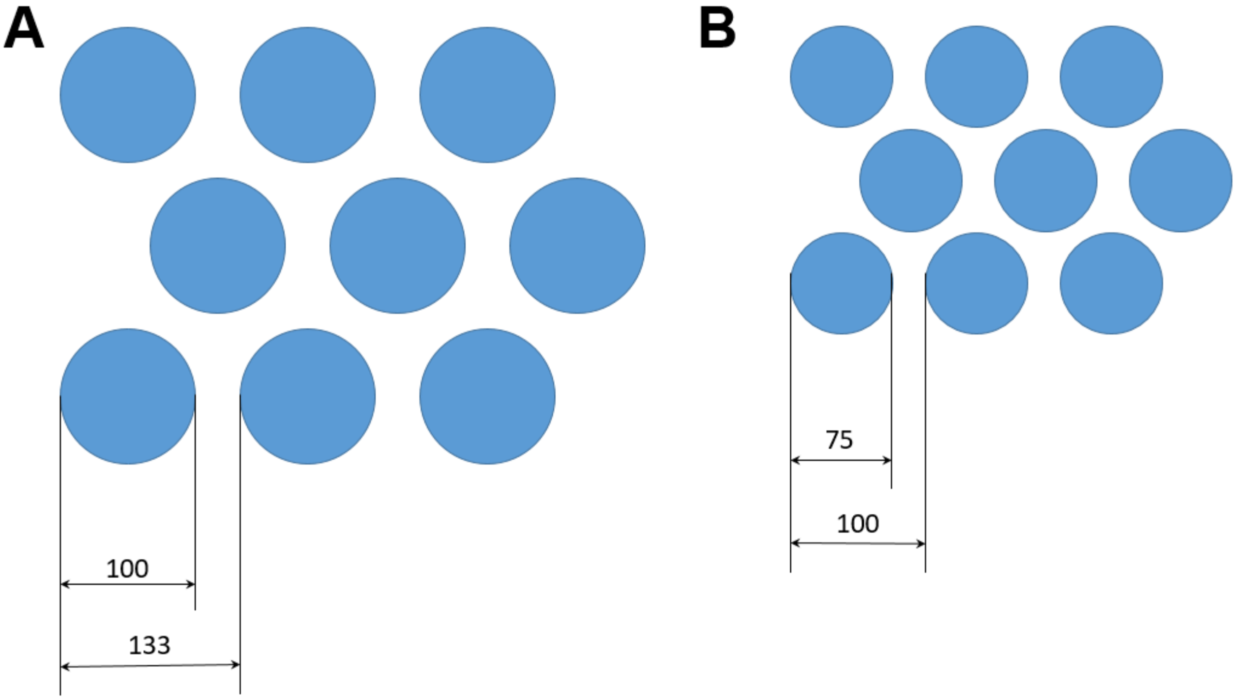

Supplementary Figure S1. Design of the pattern on the photolithography mask. The round features correspond to the dark (chromium coated) areas on the mask. **A.** 100 μm hexagonal array; **B.** 75 μm hexagonal array.

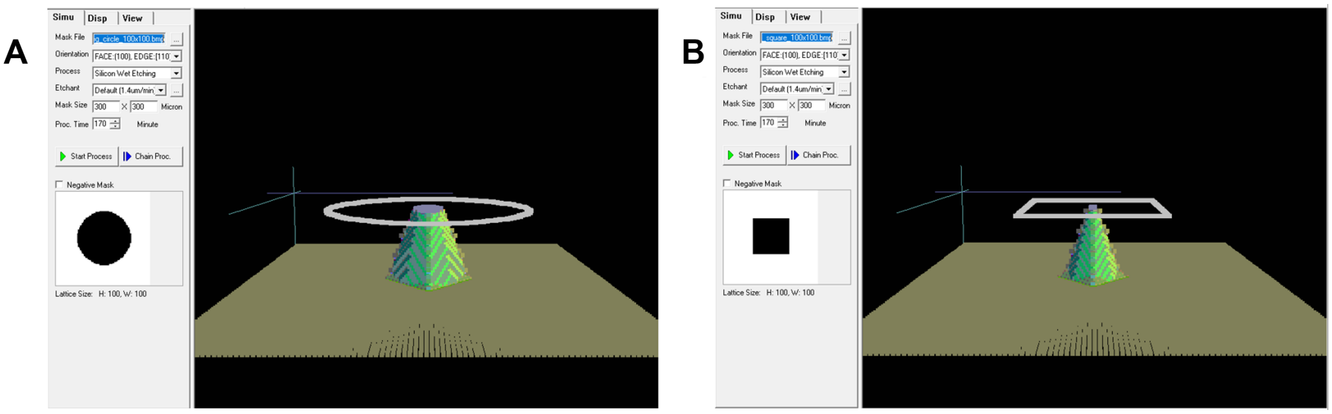

Supplementary Figure S2. Simulation results for silicon (100) etching by KOH for round (**A**) and square (**B**) mask shapes.

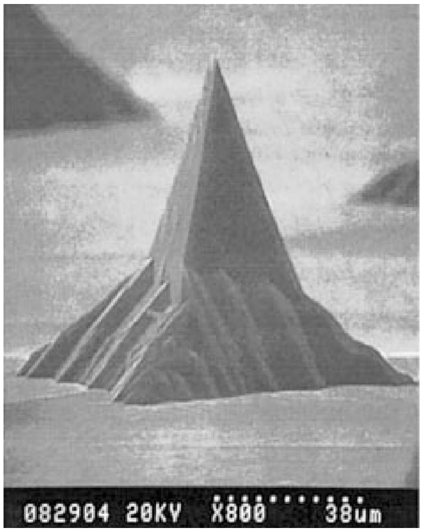

Supplementary Figure S3. Silicon pyramid as fabricated by Hashmi *et al*. SEM image from reference 2.

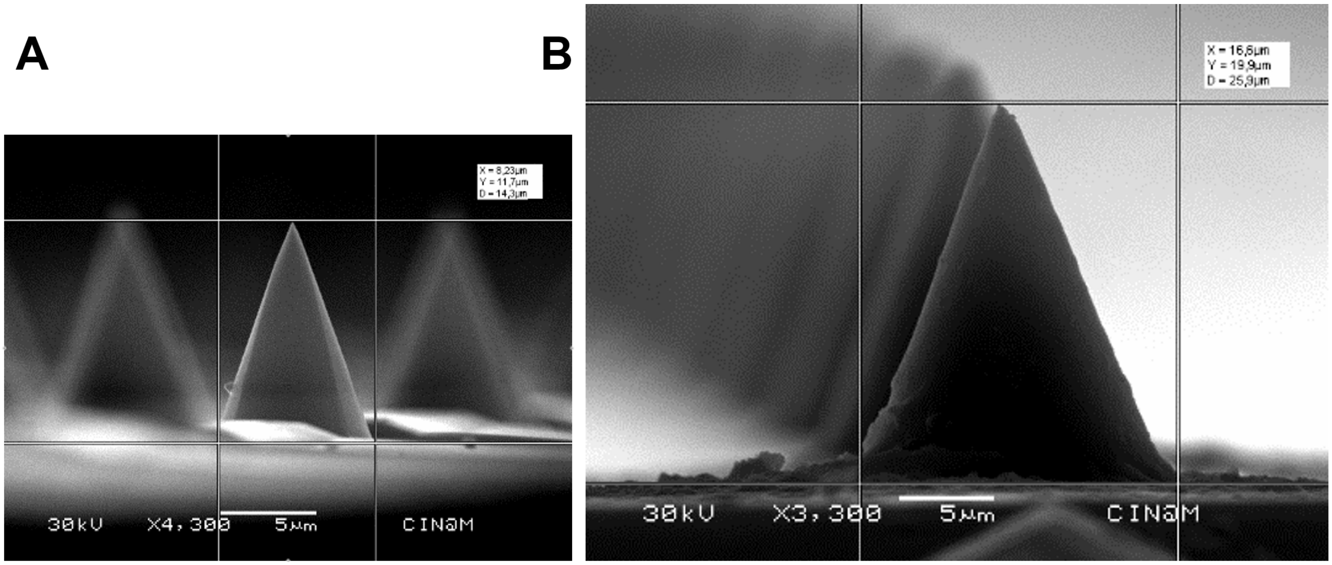

Supplementary Figure S4. Silicon pyramids of different sizes retain a similar geometry. The small (**A**) and large (**B**) pyramids are of a similar shape, with the angles at the base being 110˚ and 115˚, respectively. Manufacturing parameters: 75 µm array; 39 min. (A); 100 µm array; 70 min. (B); 400 ml fresh 45 % KOH; T=70˚C; 160 rpm.

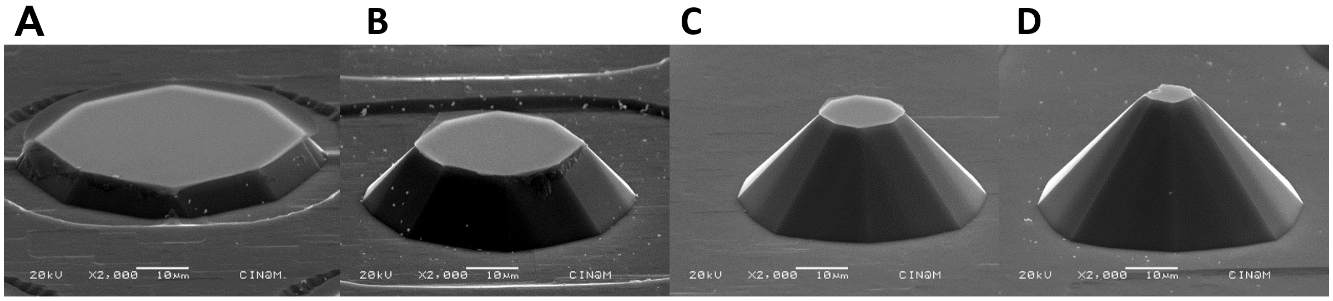

Supplementary Figure S5. SEM images of silicon pyramids in 75 µm array etched in IPA-saturated 45% KOH solution (44 ml of IPA added to 400 ml KOH solution) at T=63˚C : **A**) 25 minutes, **B**) 50 minutes, **C**) 75 minutes, **D**) 100 minutes with agitation at 170 rpm. All the samples were tilted in the SEM 70˚.

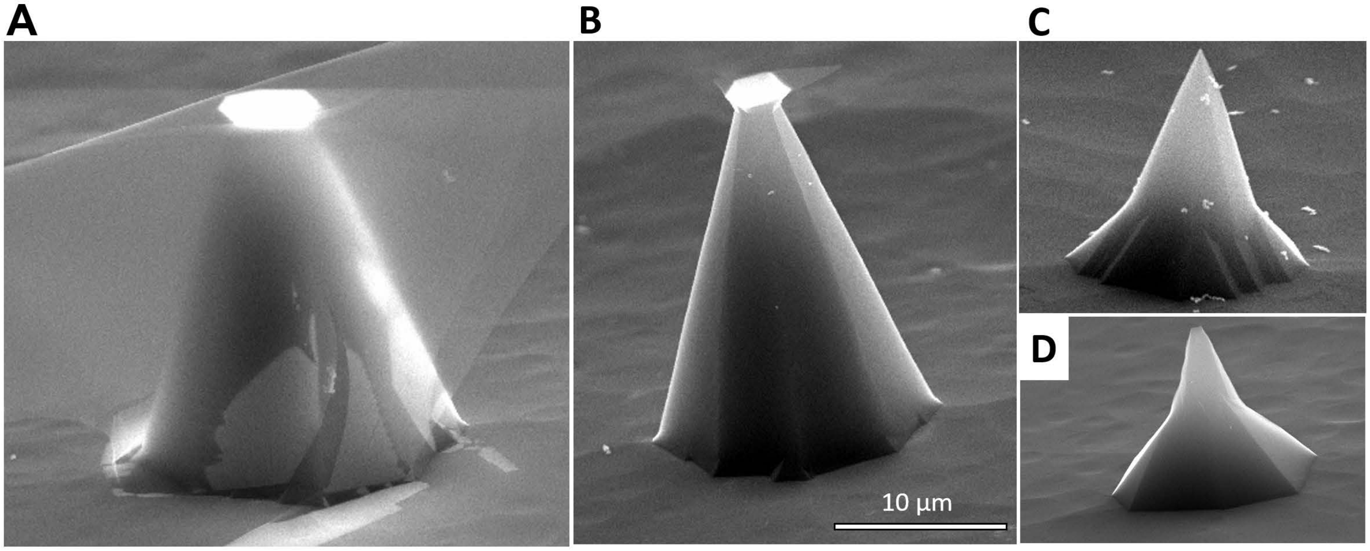

Supplementary Figure S6. SEM images of silicon pyramids in 45 μm array etched in 45% KOH solution at T=72˚C, sample holder rotation speed 170 r.p.m.: **A**) 44 minutes **B**) 45 minutes **C**) 45 minutes (followed by an additional 3 minutes, after removal and inspection with a stereomicroscope) **D**) 47 minutes (continuous). All the samples were tilted in the SEM 70˚. After 44 minutes, one can clearly see the pyramid covered by the remaining protective layer of SiO_2, with a more extensive fine, electron-transparent SiO_2 "veil". The octagonal shape of the pyramid tip is also very clearly visible. After 45 minutes of etching, the pyramid tip is much sharper than the previous one. The fine electron-transparent oxide layer lost its mechanical stability and was almost completely removed during the rinsing and drying of the sample. In order to sharpen the tips we returned the same sample to the KOH etching solution for 3 supplementary minutes (**C**). The sharp pyramids were rapidly attacked by KOH and when over-etched, only the more resistant crystalline planes close to the base remained (**D**).

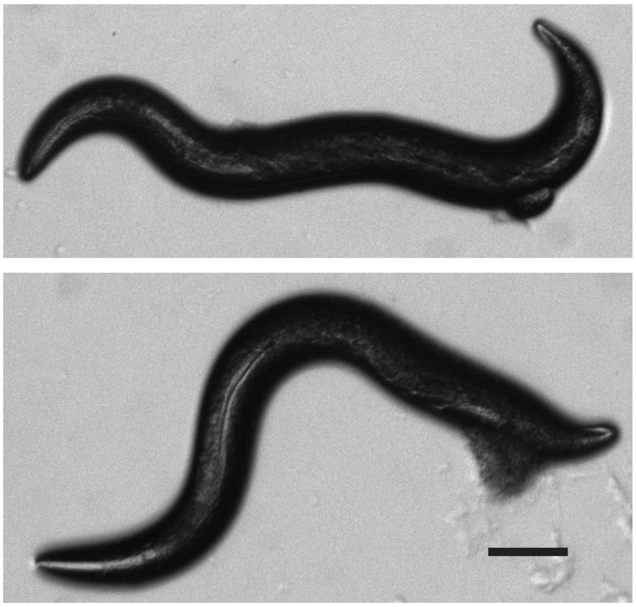

Supplementary Figure S7. Light photomicrographs of adult hermaphrodites with obvious signs of injury, 45 minutes after wounding. Scale bar 100 microns.

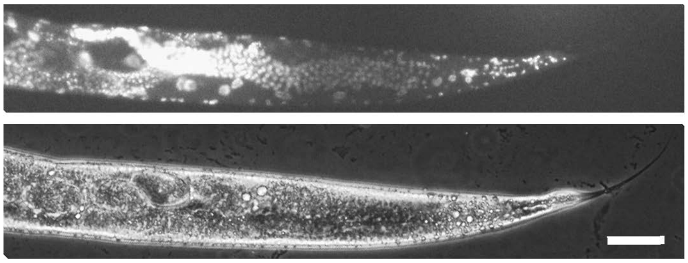

Supplementary Figure S8. Staining with DAPI of germline nuclei in injured adult hermaphrodite, as seen with fluorescence microscopy (top). The worm showed no very evident sign of wounding as judged by light microscopy (bottom). Scale bar 50 microns.

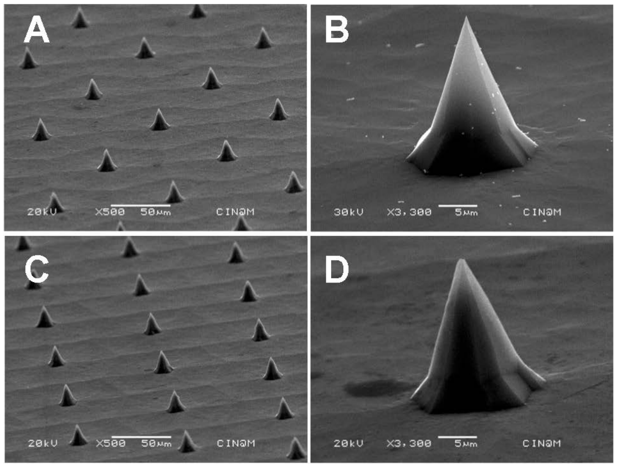

Supplementary Figure S9. SEM of an array (H75-15) before its first use (**A**, **B**) and after 160 uses (**C**, **D**). A slight wearing of the tip can be seen, but it remains extremely sharp.

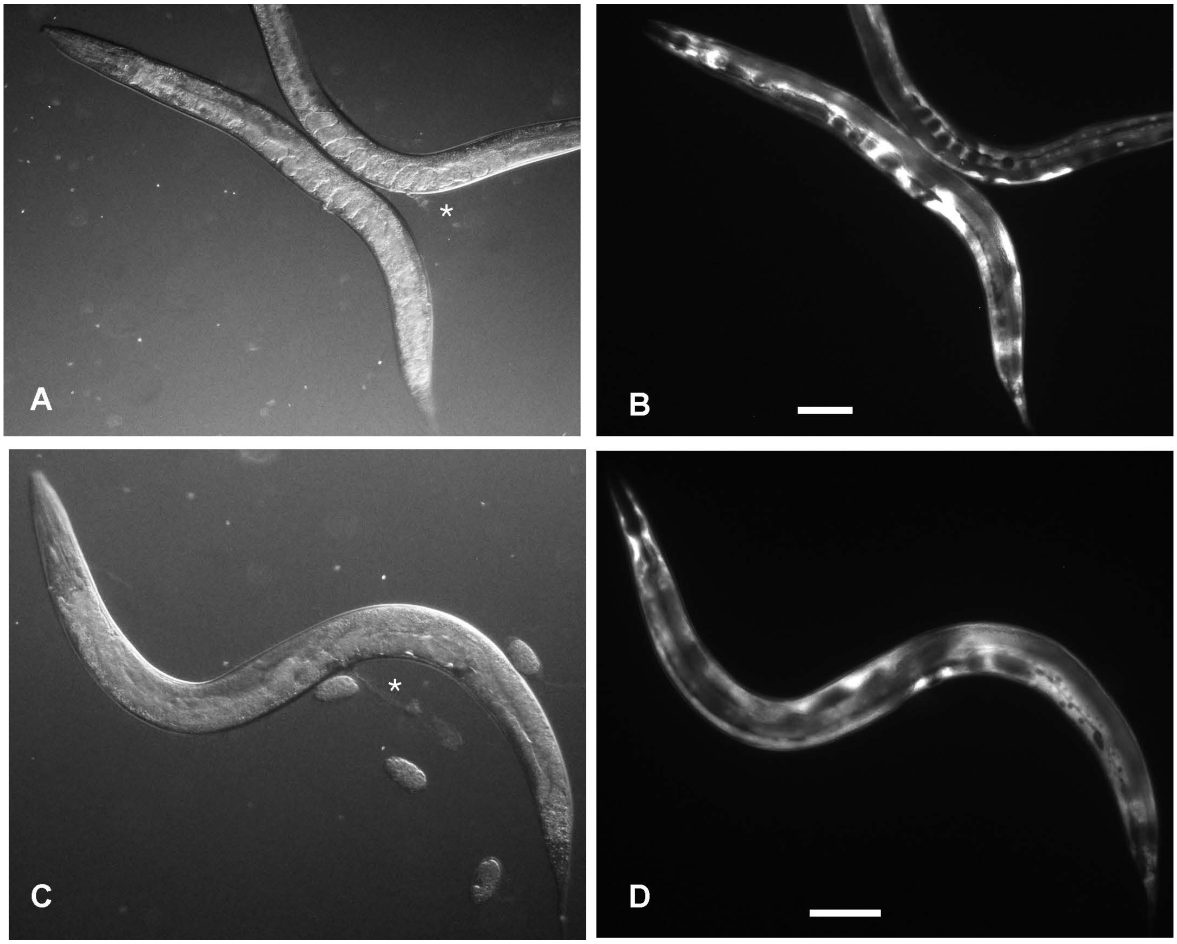

Supplementary Figure S10. Examples of IG274 worms that showed normal movement and overall morphology (as seen here with Nomarski microscopy **A, C**), with strong *nlp-29p*::GFP reporter gene expression 6 hours after wounding (**B**, **D**). The top worm in **A** and the worm in **C** appeared to leak internal contents (asterisk) when mounted for microscopy. Among normally moving worms, the vast majority (93%, n=88) exhibited no overt sign of wounding. Scale bar 100 microns.

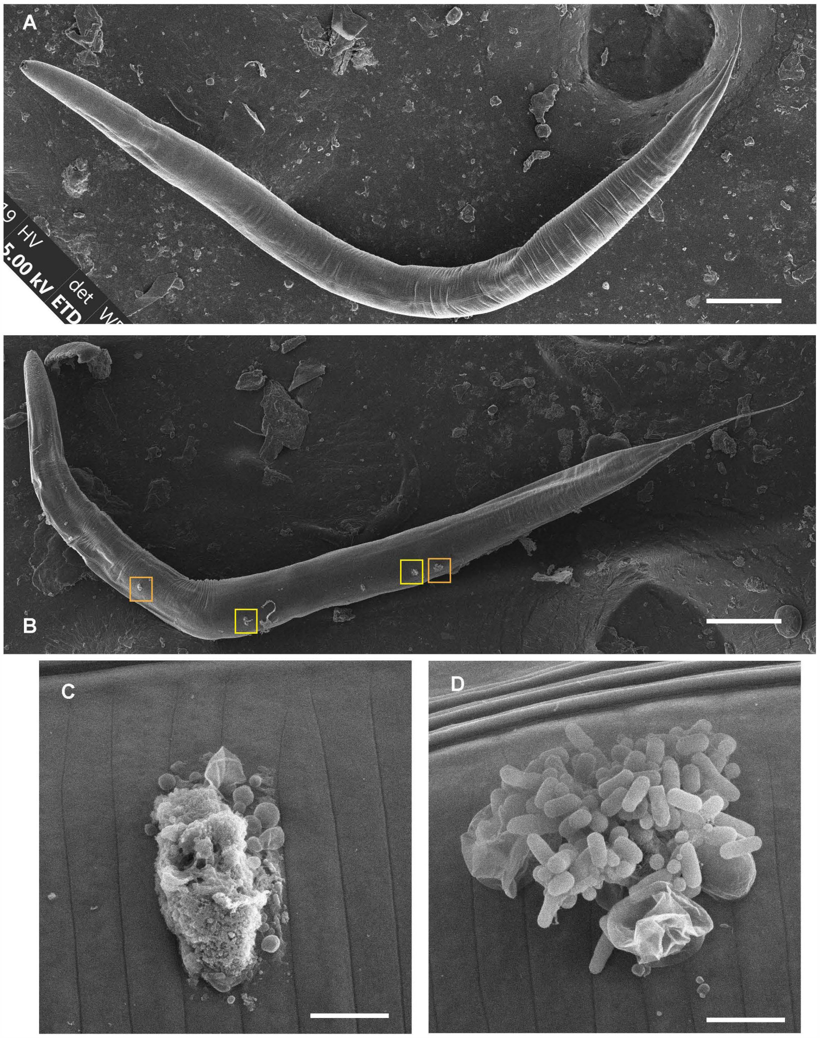

Supplementary Figure S11. SEM images of 2 worms that showed normal movement and overall morphology and had strong *nlp-29p*::GFP reporter gene expression 6 hours after wounding (**A**, **B**). The worm in **A** has not discernible injury on its visible surface. The worm in **B** has several lesions, 4 of which are boxed. Those boxed in orange are shown at higher magnification in **C** and **D**. Those boxed in yellow are shown in Figure 5D and E in the main text. Scale bar 100 microns (**A**, **B**), or 2 microns (**C**, **D**).
